# Application of artificial intelligence in laryngeal lesions: a systematic review and meta-analysis

**DOI:** 10.1007/s00405-024-09075-0

**Published:** 2024-11-22

**Authors:** Alejandro R. Marrero-Gonzalez, Tanner J. Diemer, Shaun A. Nguyen, Terence J. M. Camilon, Kirsten Meenan, Ashli O’Rourke

**Affiliations:** 1 Department of Otolaryngology-Head and Neck Surgery, Medical University of South Carolina, 135 Rutledge Avenue, MSC 550, Charleston, SC 29425 USA; 2 School of Medicine, University of Puerto Rico, San Juan, Puerto Rico; 3 University of Arizona College of Medicine, Phoenix, Phoenix, AZ USA; 4 University of South Carolina School of Medicine, Columbia, Columbia, SC USA

**Keywords:** Artificial intelligence, Larynx, Laryngology, Diagnostic tools, Endoscopy, Voice analysis, Histopathology

## Abstract

**Objective:**

The objective of this systematic review and meta-analysis was to evaluate the diagnostic accuracy of AI-assisted technologies, including endoscopy, voice analysis, and histopathology, for detecting and classifying laryngeal lesions.

**Methods:**

A systematic search was conducted in PubMed, Embase, etc. for studies utilizing voice analysis, histopathology for laryngeal lesions, or AI-assisted endoscopy. The results of diagnostic accuracy, sensitivity and specificity were synthesized by a meta-analysis.

**Results:**

12 studies employing AI-assisted endoscopy, 2 studies for voice analysis, and 4 studies for histopathology were included in the meta-analysis. The combined sensitivity of AI-assisted endoscopy was 91% (95% CI 87–94%) for the classification of benign from malignant lesions and 91% (95% CI 90–93%) for lesion detection. The highest accuracy pooled in detecting lesions versus healthy tissue was the AI-aided endoscopy was 94% (95% CI 92–97%).

**Conclusions:**

For laryngeal lesions, AI-assisted endoscopy shows excellent diagnosis accuracy. But more sizable prospective trials are needed to confirm the practical clinical value.

**Supplementary Information:**

The online version contains supplementary material available at 10.1007/s00405-024-09075-0.

## Introduction

Laryngeal lesions, which include benign and malignant conditions, represent a significant concern due to their impact on patients’ voice, swallowing and airway function, decreasing overall quality of life and health [1–4]. Currently, different methodologies are used to help differentiate between benign and malignant laryngeal lesions, including indirect laryngoscopy with or without narrow-band imaging, direct laryngoscopy, ultrasound (US), and computed tomography (CT) [5]. Many of these tools can be effective—such as laryngoscopy—but they rely heavily on a physician’s expertise, which can vary drastically depending on the physician’s experience and training [6, 7]. Studies show that less experienced clinicians may miss critical findings or misclassify lesions [6, 7]. This variability underscores the need for more standardized diagnostic tools. Other imaging modalities such as CT and MRI are essential for evaluating laryngeal lesions, particularly for assessing tumor extent and staging, but they have limitations, especially in detecting early-stage cancers [8]. CT imaging also raises concerns about radiation exposure. Considering these challenges, biopsy remains the gold standard for diagnosing laryngeal lesions, despite its inherent risks such as infection and bleeding. However, sampling errors during biopsy can lead to inaccurate or inconclusive results, further complicating the diagnostic process [9]. Artificial Intelligence (AI) technology offers the potential to overcome many of these limitations by providing automated and objective analysis of diagnostic data. Unlike human operators, AI systems can apply consistent algorithms to interpret findings, reducing the variability that arises from subjective human judgment. This consistency enhances diagnostic accuracy and could lead to more standardized care across different clinical settings [10].

AI has revolutionized healthcare, impacting disease diagnosis, treatment, and patient outcomes [11, 12]. Recent advancements in AI, particularly in machine learning and deep learning, have shown promise in enhancing the diagnostic accuracy of precancerous lesions. AI has shown significant potential in enhancing diagnostic accuracy across various medical fields. In breast cancer detection, AI systems have achieved high accuracy, often surpassing human radiologists in early tumor detection, with some studies reporting an AUC of 0.92 [13]. In lung cancer screening, AI applied to low-dose CT scans has improved the detection of pulmonary nodules and enhanced the prediction of major cardiopulmonary outcomes, offering a powerful tool for early intervention [14]. Additionally, AI systems for diabetic retinopathy have demonstrated high sensitivity and specificity, making them effective in primary care settings, leading to the authorization of such a system for widespread use [15]. These advancements indicate AI's growing role in improving diagnostic precision and accessibility in healthcare. Similar to its diagnostic utility with breast cancer and lung cancer, AI poses great potential if applied to laryngeal lesions [5]. AI has shown great promise in analyzing complex medical images and patient data to assist in early detection, classification, and treatment of laryngeal lesions [16]. For instance, different AI models have been successfully applied in analyzing endoscopic images to differentiate between benign and malignant laryngeal lesions with an accuracy of up to 93% [17]. Other applications of AI in this field include convolutional neural networks (CNNs). CNNs are a class of deep neural networks that have been successfully applied in analyzing voice changes, a possible sign of early laryngeal cancer, with a high sensitivity and specificity [18]. Using these methods AI can detect subtle imaging features that may be overlooked by experienced clinicians, leading to earlier and more accurate detection of malignancies. A study by Parker showed that machine learning via CNN-based methods to add objectivity to laryngoscopy analysis identifying small changes with high specificity [19].

The potential for AI applications to aid in the detection of laryngeal lesions is remarkable; however, the currently published literature presents a fragmented view, reporting only one AI model application per study. Previous studies focused on various individual AI application diagnostic tools, mainly on endoscopy [20]. AI has demonstrated remarkable accuracy in detecting and classifying laryngeal lesions, making it a valuable tool for early diagnosis. For example, a study by Zhou et al. highlighted that AI models applied to endoscopic images achieved a classification accuracy of over 90% in distinguishing benign from malignant laryngeal lesions, significantly aiding clinical decision-making [21]. Additionally, research by Ren et al. demonstrated that an AI-driven diagnostic system could accurately classify laryngeal lesions with an accuracy of 94%, showcasing its effectiveness in supporting physicians in making precise diagnoses [22]. Furthermore, a review by Wu et al. from 2020 to 2022 reported the recent increase in AI technology applications in otolaryngology [23]. A state-of-the-art review by Bensoussan et al. provides a comprehensive summary of current uses of AI, but it is limited by a lack of meta-analysis of their findings [24].

Since most prior investigations focus on endoscopy, our systematic review and meta-analysis included studies that detect or differentiate benign versus malignant laryngeal lesions with endoscopy, histopathology, or voice changes. This is the first systematic review that meta-analyzes several tools using AI applications in this area. The aim is to expand on the results of previous reviews and provide a broader understanding of AI's potential in this area. In recent years, AI research has experienced exponential growth. This study addresses the need for a comprehensive meta-analysis of various AI techniques by providing an up-to-date systematic review. It serves as a valuable resource that consolidates findings across diverse AI technologies, offering clinicians timely and impactful insights while efficiently summarizing the most relevant data in a single source.

## Methods

### Information source and search strategy

This study was conducted according to the Preferred Reporting Items for Systematic Reviews and Meta-Analyses (“PRISMA”) guidelines [25]. Detailed search strategies were developed using the following databases The COCHRANE Library (Wiley), PubMed (National Library of Medicine – National Institutes of Health), SCOPUS (Elsevier), and CINAHL (EBSCO). Databases were searched from inception through January 1, 2024, with the results limited to English. A combination of subject headings (e.g., Medical Subject Headings [MeSH] in PubMed) and the following keywords were used in the search: “Artificial Intelligence” or “Machine Learning,” “Laryngeal Neoplasm,” “laryngeal lesion” and “glottic neoplasm” The complete list of search terms is available in Table S1**.** Articles were screened using the review management software Covidence (Veritas Health Innovation Ltd). Titles and abstracts were screened for relevance, and full texts were reviewed to determine inclusion. References of all included articles were examined for additional studies.

### Selection criteria

The study considered several types of research designs to ensure a robust and comprehensive analysis of AI applications in diagnosing and classifying laryngeal lesions. Included study designs were cohort studies, retrospective and prospective case series, randomized controlled trials (RCTs), and case–control studies, as these designs provide valuable longitudinal data, insights into real-world clinical applications, high levels of evidence. Studies were selected if they reported key performance metrics such as accuracy, sensitivity, and specificity, which are crucial for evaluating the diagnostic utility of AI technologies. To maintain the rigor of the meta-analysis, review articles, studies with incomplete data, case reports, studies with incorrect study designs, and studies that did not measure the outcomes of interest were excluded. This exclusion was necessary to ensure that only studies contributing fully to the quantitative synthesis were included, thereby maintaining the focus on clinically relevant outcomes and the best available evidence in the application of AI for laryngeal lesion diagnosis and classification (Table 1). The study included a variety of AI approaches, specifically focusing on deep learning models such as CNNs, Recurrent Neural Networks (RNNs), and Autoencoders. Additionally, conventional machine learning models like Support Vector Machines (SVMs), Random Forests, and Naive Bayes were considered. Hybrid models, including Ensemble Learning and Transfer Learning models, were also included to capture a broad spectrum of AI applications in diagnosing and classifying laryngeal lesions. Two reviewers (A.R.M.G. and T.J.D.) independently conducted an initial screening of titles and abstracts to identify studies that met the broad eligibility criteria. This step was crucial for filtering out studies that were clearly irrelevant or did not align with the research focus. Following the initial screening, the reviewers conducted a more detailed full-text review of the remaining articles to determine their suitability for inclusion in the final analysis. This review process was guided by our PICOT framework, which focused on the following elements: Population (studies evaluating patients with laryngeal lesions using AI for diagnosis and classification), Intervention (AI technologies applied to endoscopy, voice analysis, and histopathology), Comparator (comparisons made against traditional diagnostic methods if applicable), Outcomes (sensitivity, specificity, and accuracy of AI in diagnosing and classifying laryngeal lesions), and Timing (inclusion of studies published up to January 1, 2024). To ensure best methods of the screening process, any disagreements between the two reviewers were addressed through discussion. If consensus could not be reached, a third reviewer (S.A.N.) was consulted to provide an objective assessment and resolve the conflict.

**Table 1:** Inclusion/exclusion criteria

Inclusion criteria	Exclusion criteria
(1) Patients from all ages who were assess by AI aided tool	(1) Studies that lack variables for meta-analysis
(2) Studies that included sensitivity, specificity, or accuracy	(2) Studies that used animal studies
(3) Studies that assess detection of laryngeal lesion versus healthy tissue using AI aided technology	(3) Studies those asses other site lesion such as oral cavity, etc
(4) Studies that assess classification of benign vs malignant lesion using AI aided technology	

### Data collection process and data items

The two reviewers independently extracted data from each included study to ensure accuracy and minimize the risk of bias. This process involved systematically gathering information on various study characteristics, including the author names, publication year, and participant demographics, such as age, gender, and clinical context. Additionally, the specific AI model utilized in each study (e.g., CNNs, RNNs, or SVMs) was documented, along with key performance metrics like accuracy, sensitivity, and specificity. After the initial data extraction, the reviewers compared their results to identify any discrepancies. In cases where disagreements arose, a third reviewer (S.A.N.) was consulted to provide an objective resolution.

### Critical appraisal

The included articles were critically appraised to assess the level of evidence using the Oxford Center for Evidence-Based Medicine criteria [26]. Since the included studies were a mix of randomized and non-randomized studies, QUADAS-2 was used. QUADAS-2 is a risk-of-bias tool that assesses four domains. These four domains are patient selection, index test, reference standard, and flow and timing. The patient selection domain examines how participants were chosen, ensuring they represent the typical clinical population. The index test domain evaluates whether the AI technology was applied and interpreted independently of the reference standard, maintaining objectivity. The reference standard domain assesses the validity and application of the "gold standard" diagnostic method without bias from the index test results. Finally, the flow and timing domain looks at the sequence and timing of tests to ensure consistency and reduce potential bias from variations in test administration. Each domain is assessed for risk of bias, and the first three domains are also evaluated for concerns about applicability [27]. Two authors (A.R.M.G and T.J.D.) independently performed risk assessments on all studies. The risk of bias for each aspect was graded as low, unclear, or high. The third reviewer (T.C) resolved any conflicts between reviewers.

### Data analysis and synthesis of results (statistical analysis)

Meta‐analysis of continuous measures (sensitivity, specificity, and accuracy) was performed with Cochrane Review Manager (RevMan) version 5.4 (The Cochrane Collaboration 2020, United Kingdom). A meta-analysis of proportions (gender) was performed using MedCalc 22.017 (MedCalc Software, Ostend, Belgium). Each measure (mean [log(mean0] / proportion (%) and 95% confidence interval (CI)) was weighted according to the number of patients affected. Heterogeneity among studies was assessed using χ^2^ and I^2^ statistics with fixed effects (I^2^ < 50%) and random effects (I^2^ > 50%). We conducted a meta-regression where we examined factors influencing diagnostic accuracy, including the number of images, and AI model type using R (software 4.4.1). In addition, potential publication bias was evaluated by visual inspection of the funnel plot and Egger’s regression test, which statistically examines the asymmetry of the funnel plot [28, 29]. A *p* value of < 0.05 was considered to indicate a significant difference for all statistical tests.

## Results

### Overview of search strategy

**Fig. 1:**
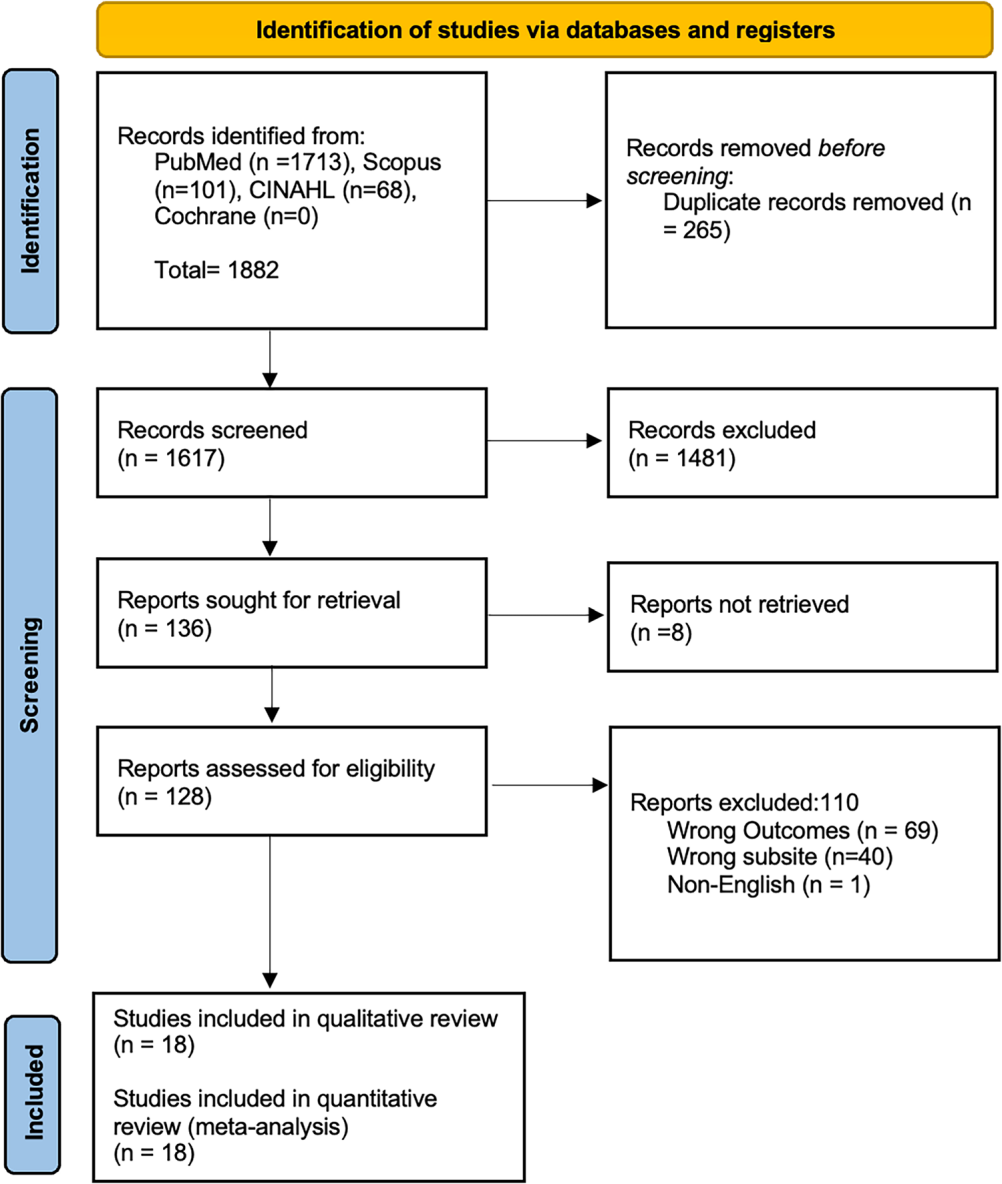
PRISMA 2020 flow diagram for new systematic reviews which included searches of databases and registers only

A comprehensive literature search found 1713 unique articles related to AI and laryngeal pathology. Title and abstract review led to the exclusion of 1558 articles, resulting in 155 studies assessed in full text. After careful consideration, 18 articles were included in the systematic review and the meta-analysis. The search and screening process is in Fig. 1, which shows the PRISMA diagram [30]. The studies included non-randomized cohort studies, which were classified as level 3 based on the Oxford level of evidence and were published from the database's inception to January 2024.

### Risk of bias

Critical appraisal of studies indicated an acceptably low risk of bias for all included studies. Potential sources of bias in non-randomized studies with QUADAS-2 (Fig. S1) were most pronounced due to the index test, selection of participants, and reference standard. A funnel plot with Egger’s test (Fig. S2) (– 2.7, 95% CI – 9.6 to 4.1, p = 0.33) indicated that all studies were within the funnel, suggesting little publication bias.

### Overview of included studies

The descriptive features of the included studies are summarized in Table 2 [18, 21, 22, 31–45]. Eighteen studies comprising 18,944 patients were included, with the proportion of males being 92% (95% CI 85.6–96.6%). Of the 18 studies included in the meta-analysis, 12 (66.7%) reported AI-aided laryngeal endoscopy [22, 31–41], 2 (11.1%) reported detection of lesions using voice changes [18, 45], and 4 (22.2%) reported classification of benign versus malignant using histopathology [21, 42–44]. The number of samples/images used in the AI models ranges from 124 to 24,667, totaling 115,136.

**Table 2:** Characteristics of included studies

Author Year	Study design (oxford level of evidence)	No. patients	No. images (total)	Lesion site	Aims and outcomes	Reference standard
*Endoscopy*						
Bengs 2020	Retrospective (3)	100	–	Larynx	Discrimination of normal tissue and cancer	Histopathology
Cho 2021	Retrospective (3)	4106	4106	Larynx	Classification into normal tissue, polyps, nodules, leukoplakia, cysts, papillomas, Reinke’s edema, granulomas, and vocal cord palsies	Histopathology and ENT-specialist interpretation
Dunham 2020	Retrospective (3)	–	19,353	Larynx	Discrimination of benign and malignant/premalignant	Histopathology and ENT-specialist interpretation
Esmaeili 2019	Retrospective (3)	32	1485	Larynx	Discrimination of normal and malignant tissue	Histopathology and ENT-specialist interpretation
Esmaeili 2021	Retrospective (3)	146	8181	Larynx	Discrimination of normal and malignant tissue	Histopathology
Inaba 2020	Retrospective (3)	374	2400	Larynx	Discrimination of normal and malignant tissue	Histopathology
Moccia 2017	Retrospective (3)	33	1320	Larynx	Classification of normal tissue, leukoplakia	Histopathology
Ren 2020	Retrospective (3)	9231	24,667	Larynx	Classification of normal tissue, polyps, nodules, leukoplakia, and cancer	Histopathology and ENT-specialist interpretation
Turkmen 2015	Retrospective (3)	70	124	Larynx	Classification of normal tissue, polyps, nodules, laryngitis, and sulcus vocalis	ENT-specialist interpretation
Wu 2021	Retrospective (3)	–	10,760	Larynx	Discrimination of normal and malignant tissue	Histopathology
Xiong 2019	Retrospective (3)	2208	14,897	Larynx	Classification of cancer, precancerous lesions, benign tumors, and normal tissue	Histopathology
Yan 2023	Retrospective (3)	2179	2179	Larynx	Discrimination of normal and malignant tissue	ENT-specialist interpretation
*Histopathology*						
Li 2022	Retrospective (3)	10	414	Larynx	Discrimination of normal and malignant tissue	Histopathology
Valjarevic 2023	Retrospective (3)	100	2000	Larynx	Discrimination of normal and malignant tissue	Histopathology
Zhang 2019	Retrospective (3)	78	18,750	Larynx	Discrimination of normal and malignant tissue	Histopathology
Zhou 2021	Retrospective (3)	10	4500	Larynx	Discrimination of normal and malignant tissue	Histopathology
*Voice pathology*						
Kim 2020	Retrospective (3)	95	–	Larynx	Classification of normal voice and pathological voice	Histopathology, ENT-specialist interpretation
Wang 2022	Retrospective (3)	172	–	Larynx	Classification of normal voice and pathological voice	Histopathology

### Laryngeal lesion detection

**Fig. 2:**
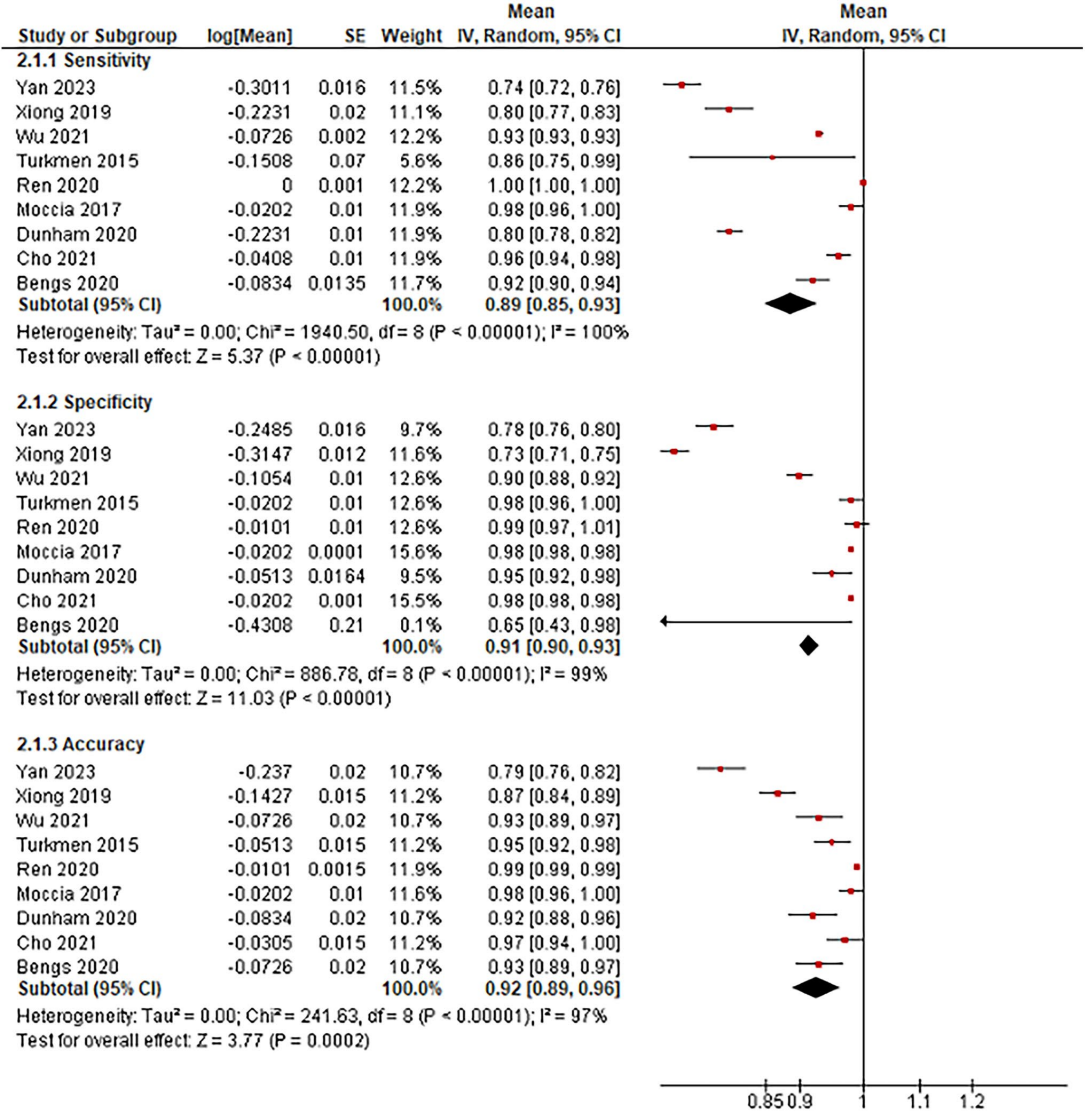
Forest plot reporting AI-aided endoscopy's sensitivity, specificity, and accuracy in identifying health tissue versus laryngeal lesions

Nine studies were identified that used AI application in laryngeal endoscopy to detect lesions and included sensitivity, specificity, and accuracy. Figure 2 shows a pooled analysis of the outcomes of AI application in laryngeal endoscopy for detecting lesions versus healthy tissue. AI application in laryngeal endoscopy showed a pooled mean sensitivity and specificity were 89% (95% CI 85–93%) and 91% (95% CI 90–93%). The pooled accuracy between all studies was 92% (95% CI 89–96%). The mean number of images the AI-aided endoscopy used in this modality was 8765.66.

**Fig. 3:**
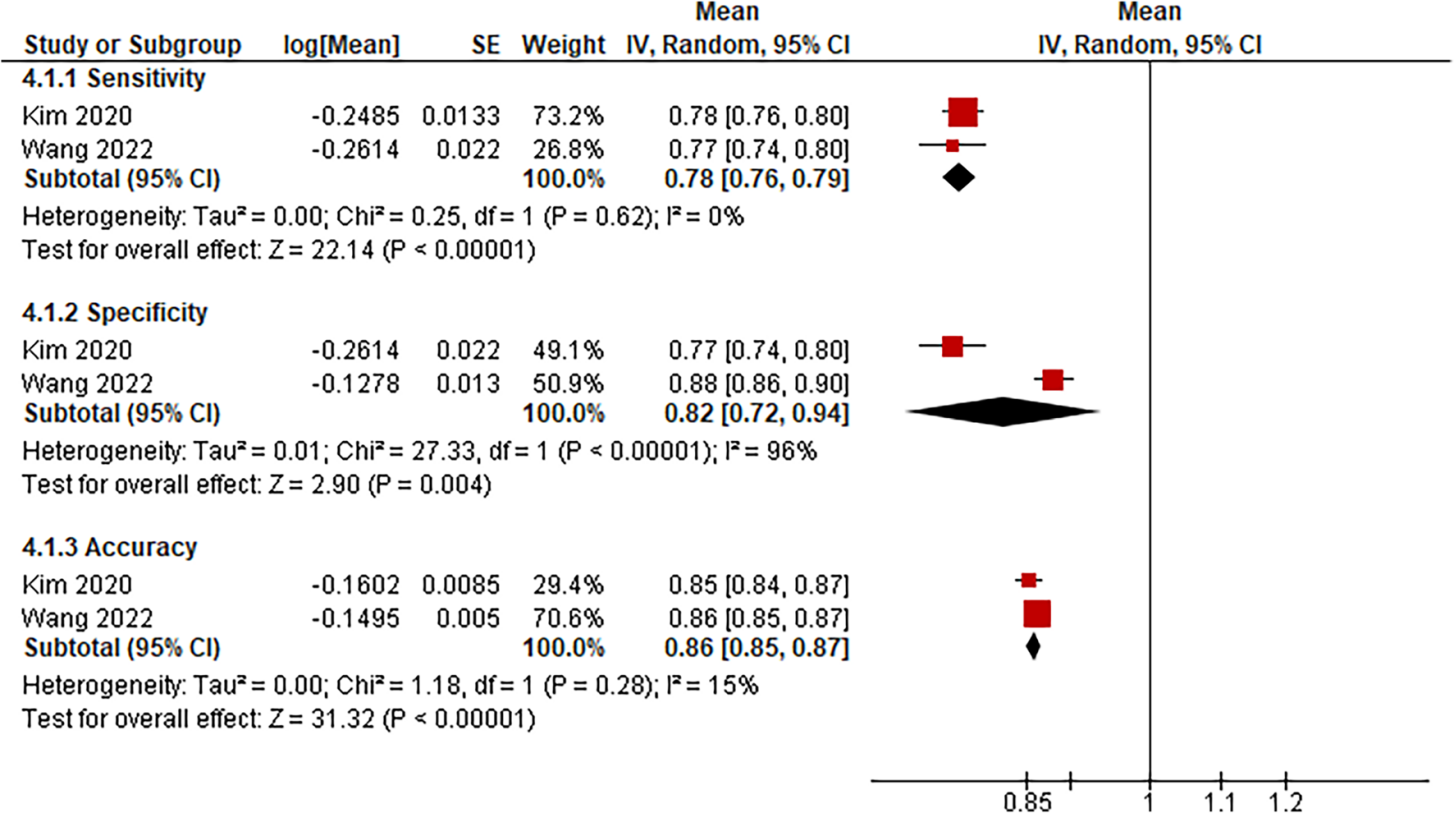
Forest plot reporting the sensitivity, specificity, and accuracy of AI-aided voice analysis program on identifying health tissue versus laryngeal lesions

This meta-analysis included only one other AI-assisted diagnostic tool, which was voice-analysis software, for detecting possible laryngeal lesions. Other investigations with diagnostic tools such as CT were identified in the search but were excluded due to their high risk of bias and lack of complete outcomes for meta-analysis. Voice analysis’ pooled mean sensitivity and specificity were 78% (95% CI 76–79%) and 82% (95% CI 72–94%). The pooled accuracy between all studies was 86% (95% CI 85–87%) (Fig. 3).

### Classification of benign versus malignant lesions

**Fig. 4:**
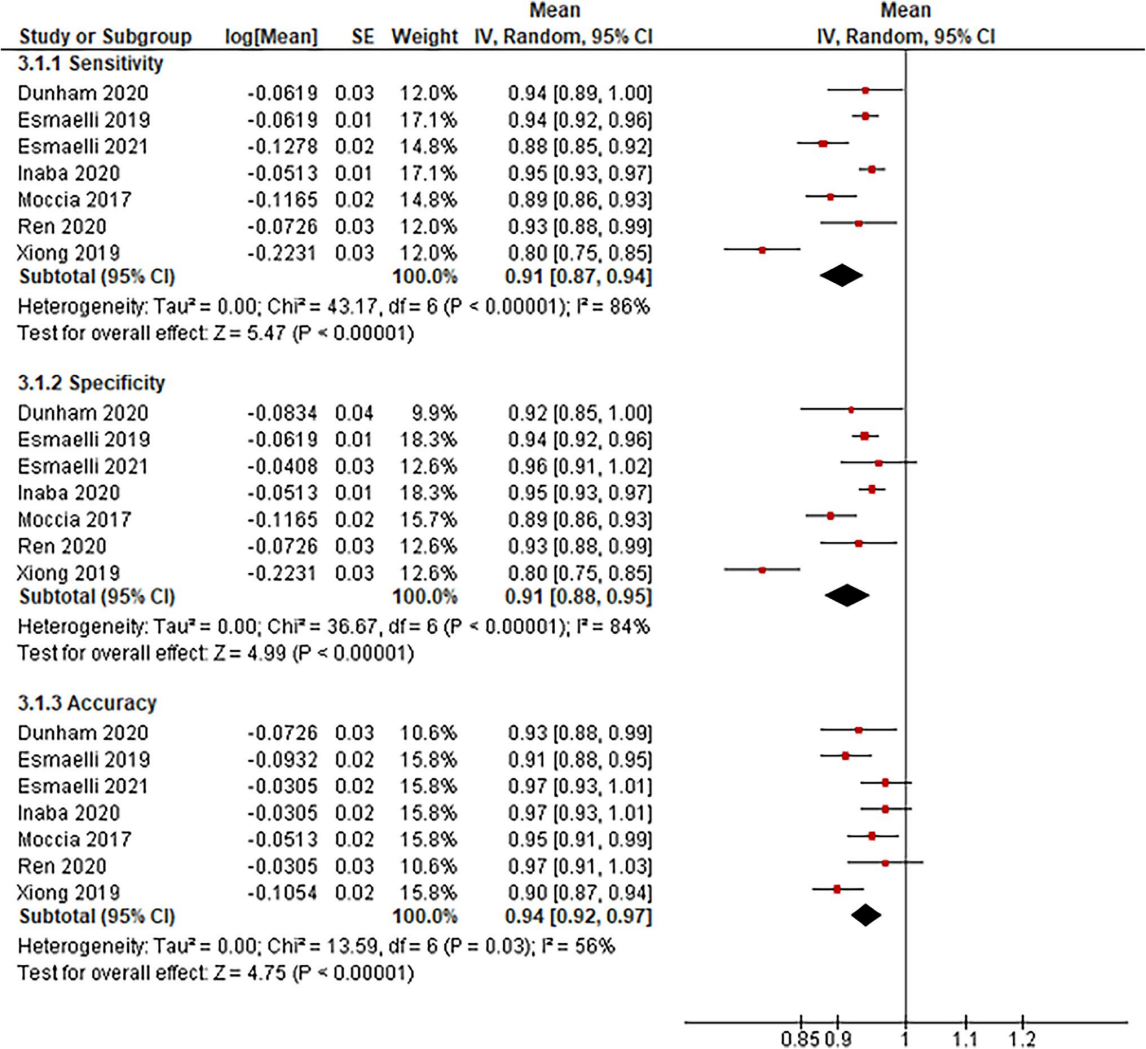
Forest plot reporting AI-aided endoscopy's sensitivity, specificity, and accuracy in classifying benign versus malignant laryngeal lesions

Seven studies were identified that used AI application in laryngeal endoscopy to differentiate benign versus malignant lesions, including sensitivity, specificity, and accuracy. Figure 4 shows a pooled analysis of sensitivity, specificity, and accuracy for this diagnostic tool. The pooled mean sensitivity and specificity for laryngeal endoscopy distinguishing between lesions were 91% (95% CI 87–94%) and 91% (95% CI 88–95%), respectively. The pooled accuracy between all studies was 94% (95% CI 92–97%). The mean number of images the AI model used was 8133.33.

**Fig. 5:**
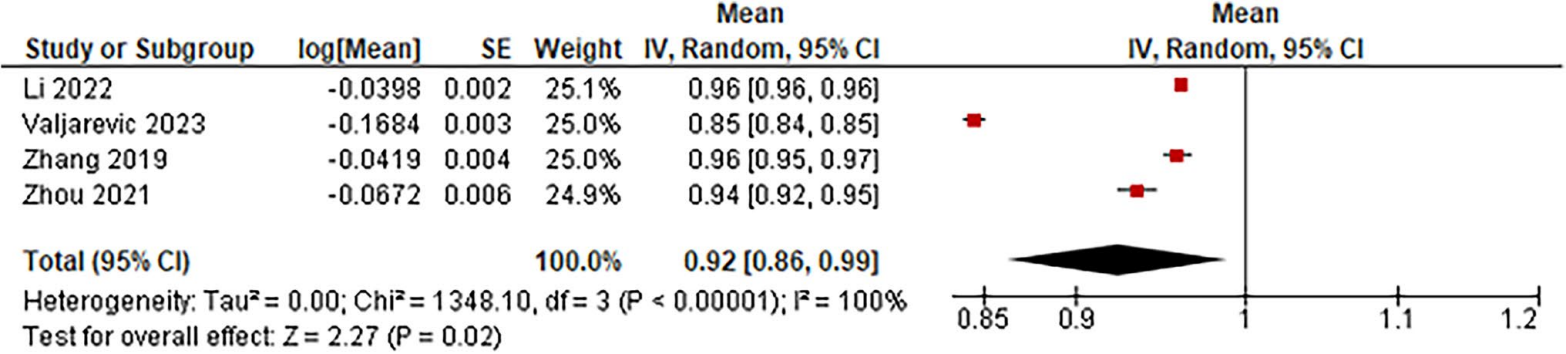
Forest plot reporting the accuracy of AI-aided pathology in classifying benign versus malignant laryngeal lesions

AI-aided histopathology classification was the only diagnostic tool that used AI to differentiate benign versus malignant laryngeal lesions. Four studies reported using AI to aid in the histopathology classification, but the only outcome that could be analyzed in these studies was accuracy due to the lack of standard deviation or p-value in other outcomes, such as sensitivity and specificity. Figure 5, the pooled accuracy between these studies was 92% (95% CI 86–99%). The mean number of images the AI model used was 6,416, ranging from 414 to 18,750, with a total of 25,664.

### Meta-regression analysis

The meta-regression analysis demonstrated a significant relationship between diagnostic accuracy and the AI technique used, with an R-squared value of 91.5%, indicating that 91.5% of the variability in diagnostic accuracy is explained by the type of AI modality (endoscopy, voice analysis, or histopathology). After adjusting for the number of predictors, the adjusted R-squared remained high at 87.2%, further supporting the strength of this relationship. The model was statistically significant (F-statistic = 21.49, p = 0.0435), suggesting that the choice of AI technique significantly influences diagnostic accuracy. Specifically, histopathology-based AI techniques were associated with a 24.46% higher diagnostic accuracy compared to endoscopy, as indicated by a coefficient of 0.2446 (95% CI 0.018 to 0.472, p = 0.044). However, due to limited data, it was not possible to determine the effect of voice analysis on diagnostic accuracy in this model.

## Discussion

### Main findings

Laryngeal cancer is one of the most common subtypes of head and neck cancer, with an estimated 12,380 new cases per year [46]. According to the American Cancer Society, the 5-year relative survival rate for glottic cancer for all stages is 77%, but when stratified, it ranges from 84% for localized disease to 45% for distant disease [46]. This decline in survival with distant disease highlights the importance of early detection and accurate diagnosis of laryngeal lesions.

There has been an exponential increase in published research on AI applications in healthcare in the past decade [47, 48]. Most studies have shown high efficacy in the detection of lesions in other body sites, such as the colon and breast. This systematic review and meta-analysis supports that AI could be useful in laryngology, improving early detection and the accuracy of diagnosis of laryngeal lesions. Of the included studies, all AI models had very high sensitivity, specificity, and accuracy. The accuracy of the included studies ranges from 86 and 94%. AI application in laryngeal endoscopy had the highest accuracy with a pooled mean of 92% (95% CI 89–96%). This is supported by previous studies that reported the accuracy of AI used in laryngeal endoscopy was between 80 and 99% [20]. These results show that AI applications have great efficacy and the potential to be introduced as diagnostic tools to aid physicians. Given the accessibility of laryngoscopy as an office procedure, implementing AI into this diagnostic tool may help improve diagnosis and early detection of malignant lesions, which could potentially lead to improved outcomes [49, 50].

Other techniques also show promising results, but a lack of standardized outcomes makes it challenging to compare diagnostic tools. The accuracy of reference standard tests, such as histopathology and expert-reviewed imaging, is generally high, but it is not without limitations. Variability in interpretation, especially in imaging, can introduce inconsistencies, which AI technologies aim to mitigate by offering more objective and standardized assessments. The finding of our meta regression suggests that while histopathology-based AI models are highly effective for diagnosing laryngeal lesions, further research is needed to better evaluate the impact of voice analysis and other contextual factors on diagnostic accuracy. A study by Kim et al. explored AI-assisted voice analysis programs to identify healthy tissue versus laryngeal cancer accurately and showed promising results, with 15% more accurate detection when compared with laryngologists [51]. Our review of voice-analysis studies supports these findings, with meta-analysis showing a mean pooled accuracy of 86%

Even though identifying laryngeal lesions is important, differentiating between benign and malignant lesions is crucial for patient outcomes. AI-aided technology has also shown great potential in this area. A study by Hu et al. reported that AI-aided ultrasound correctly classified benign versus malignant nodules in the liver with an accuracy of 91% [52]. This AI model even outperformed the diagnostic accuracy of radiology residents and matched that of experts [52]. Other studies support these findings in malignant thyroid, breast, and lung lesions [53–55]. Our study also supports that AI application in diagnostic tools such as endoscopy is useful in differentiating benign versus malignant lesions with an accuracy of up to 94%. These results further support the utility of integrating AI into these commonly used diagnostic tools such as laryngeal endoscopy. A study by Ren et al., which used the largest dataset of images of all included studies, reported that compared to expert physicians, AI has better overall accuracy in the classification of leukoplakia (65% vs 91%) and glottic cancer (54% vs 90%) [22]. This shows potential for integrating AI into clinical practice, especially for the future generation of physicians.

In summary, our finding reported the diagnostic accuracy of AI technologies in laryngeal lesion detection varies across modalities. AI-assisted endoscopy demonstrated the highest diagnostic performance, with a pooled sensitivity of 91% (95% CI 87–94%) for differentiating benign from malignant lesions and a pooled accuracy of 94% (95% CI 92–97%) for lesion detection. Histopathology-based AI models, although less frequently studied, also achieved a high pooled accuracy of 92% (95% CI 86–99%). In contrast, voice analysis, while promising, showed a relatively lower pooled sensitivity of 78% (95% CI 76–79%) and an accuracy of 86% (95% CI 85–87%). These results indicate that while endoscopy and histopathology show strong diagnostic potential for lesion classification, voice analysis may require further refinement and validation to match the efficacy of imaging-based AI technologies.

### Applicability of AI in the clinical setting

The results of this meta-analysis suggest that AI is a diagnostic tool that could be valuable for assessing laryngeal lesions. A previous systematic review reported that AI has high accuracy and clinical utility when assessing images of laryngeal lesions [20]. This raises the question of who would benefit the most from AI in the clinical setting. The most significant utility of AI currently is in the classification of benign versus malignant lesions [56]. A review by Sampieri et al. found that AI models perform better in binary classification of benign vs malignant but lose accuracy in multiclass classification [56]. Of the included studies, Dunham et al. reported this finding, showing 93% accuracy when the model classified between benign and malignant but dropping to 83% when classifying between several different lesions [57]. This is similar to humans; the more complicated the task, the lower the accuracy. However, it should be noted that the research studies presented so far have focused solely on the research-oriented setting [56]. As real-world clinical applications of this technology have yet to be studied, it is difficult to assess the actual implications of this technology in a clinical setting. AI technologies could be particularly beneficial for patients in rural or underserved areas, where access to specialist care is limited. Moreover, busy clinical environments could also benefit from the enhanced efficiency and diagnostic consistency that AI tools offer. Several barriers exist before this technology is helpful in clinical practice, such as the AI software used is not commercially available. Significant obstacles to clinical implementation also include the current lack of regulatory clearance and commercial availability of AI tools. Additionally, the integration of these technologies will necessitate updated equipment and comprehensive training programs to ensure healthcare professionals can effectively utilize and interpret AI-generated results. In addition, AI implementation in healthcare must address significant privacy concerns, as these models depend on large datasets containing sensitive patient information. Additionally, potential biases in the data used to train AI models can result in inequitable outcomes, particularly for underrepresented patient populations.

A study by Alowais et al. reported that integrating AI into healthcare improves disease diagnosis, but it does come with challenges. Incorporating AI into clinical practice could bring problems with data privacy and bias [58]. More studies that show standardized outcomes are needed to assess the potential for AI-aided diagnostic and classification tools in laryngology. In addition, prospective studies that implement AI-aided laryngeal endoscopy systems in clinical practice are essential.

### Limitations

It is important to consider several limitations when interpreting the results of this study. First, despite a comprehensive literature search, publication bias is possible. This could lead to overestimating AI’s effectiveness in detecting and classifying laryngeal lesions. Second, using different AI models and diagnostic tools across the included studies may limit the generalizability of our findings. Different AI algorithms, training datasets, and validation methods can significantly impact the results, making direct comparisons difficult. Finally, standardized reporting on the technical details of AI models, such as architecture, training parameters, and data augmentation techniques, needs to be improved. This limits the reproducibility of the studies and hinders the assessment of the utility of AI applications in laryngology.

The study has a notable limitation due to the high heterogeneity among the included studies. Study heterogeneity poses a significant challenge in our meta-analysis, arising from differences in study design, patient populations, and AI model types. This variability limits the generalizability of our findings and underscores the importance of standardizing research protocols in future studies. Apart from heterogeneity, this study faced limitations due to the prevalence of retrospective designs and small sample sizes, which constrain the robustness of our conclusions. The potential for publication bias, especially the underreporting of negative outcomes, may further skew the perceived effectiveness of AI technologies. Additionally, the lack of sufficient data on various AI algorithms and architectures limited our ability to fully assess and compare their effectiveness. It may affect the applicability of the results and could affect the actual effectiveness of AI tools in diagnosing and classifying laryngeal lesions in various clinical settings.

In summary, while our findings suggest that AI applications have the potential to diagnose and classify laryngeal lesions, these limitations highlight the need for further research. Although the AI models reviewed in this study exhibit high accuracy, further validation is needed before they can be widely adopted in clinical practice. Additional studies are required to assess the performance of these models in real-world settings to ensure their robustness and reliability. Future research should include large, multicenter studies to validate these findings across diverse patient populations. Comparative effectiveness studies are also crucial to directly compare AI-assisted diagnostics with traditional methods, thereby determining their true clinical value.

## Conclusion

The study reports high accuracy, sensitivity, and specificity of AI-aided tools, especially in laryngeal endoscopy, for classifying benign and malignant lesions. These findings indicate that AI could enhance early diagnosis and classification, potentially improving patient outcomes. However, several limitations highlight the need for further research to validate and refine AI applications in laryngology.

## Supplementary Information

Below is the link to the electronic supplementary material.

Supplementary file1 (DOCX 1011 KB)

## Data Availability

The data that support the findings of this study are available from the corresponding author upon reasonable request.

## References

[1] Bergström L, Ward EC, Finizia C (2016) Voice rehabilitation for laryngeal cancer patients: Functional outcomes and patient perceptions. Laryngoscope 126(9):2029–2035. 10.1002/lary.25919 27010512 10.1002/lary.25919

[2] Anschuetz L, Shelan M, Dematté M, Schubert AD, Giger R, Elicin O (2019) Long-term functional outcome after laryngeal cancer treatment. Radiat Oncol 14(1):101. 10.1186/s13014-019-1299-8 31186027 10.1186/s13014-019-1299-8 PMC6558792

[3] Tuomi L, Karlsson T (2020) Voice quality, function, and quality of life for laryngeal cancer: a prospective longitudinal study up to 24 months following radiotherapy. Ear Nose Throat J 100(10_suppl):913S-920S. 10.1177/0145561320929941 32484410 10.1177/0145561320929941

[4] Mansour MMH, Abdel-Aziz MF, Saafan ME, Al-Afandi HRM, Darweesh M (2016) Voice, swallowing, and quality of life after management of laryngeal cancer with different treatment modalities. Egypt J Otolaryngol 32(1):37–44. 10.4103/1012-5574.175845

[5] Alonso-Coello P, Rigau D, Sanabria AJ, Plaza V, Miravitlles M, Martinez L (2013) Quality and strength: the GRADE system for formulating recommendations in clinical practice guidelines. Arch Bronconeumol 49(6):261–267. 10.1016/j.arbres.2012.12.001 23434203 10.1016/j.arbres.2012.12.001

[6] Żurek M, Jasak K, Niemczyk K, Rzepakowska A (2022) Artificial intelligence in laryngeal endoscopy: systematic review and meta-analysis. J Clin Med. 10.3390/jcm11102752 35628878 10.3390/jcm11102752 PMC9144710

[7] Unger J, Lohscheller J, Reiter M, Eder K, Betz CS, Schuster M (2015) A noninvasive procedure for early-stage discrimination of malignant and precancerous vocal fold lesions based on laryngeal dynamics analysis. Can Res 75(1):31–39 10.1158/0008-5472.CAN-14-1458 25371410

[8] Wu JH, Zhao J, Li ZH et al (2016) Comparison of CT and MRI in diagnosis of laryngeal carcinoma with anterior vocal commissure involvement. Sci Rep 6:30353. 10.1038/srep30353 27480073 10.1038/srep30353 PMC4969597

[9] Fleskens SAJHM, Bergshoeff VE, Voogd AC et al (2011) Interobserver variability of laryngeal mucosal premalignant lesions: a histopathological evaluation. Mod Pathol 24(7):892–898. 10.1038/modpathol.2011.50 21499237 10.1038/modpathol.2011.50

[10] Najjar R (2023) Redefining radiology: a review of artificial intelligence integration in medical imaging. Diagnostics (Basel). 10.3390/diagnostics13172760 37685300 10.3390/diagnostics13172760 PMC10487271

[11] Noorbakhsh-Sabet N, Zand R, Zhang Y, Abedi V (2019) Artificial intelligence transforms the future of health care. Am J Med 132(7):795–801 30710543 10.1016/j.amjmed.2019.01.017 PMC6669105

[12] Matheny ME, Whicher D, Israni ST (2020) Artificial intelligence in health care: a report from the National Academy of Medicine. JAMA 323(6):509–510 31845963 10.1001/jama.2019.21579

[13] Witowski J, Heacock L, Reig B et al (2022) Improving breast cancer diagnostics with deep learning for MRI. Sci Transl Med 14(664):eabo4802. 10.1126/scitranslmed.abo4802 36170446 10.1126/scitranslmed.abo4802 PMC10323699

[14] Chamberlin JH, Kocher MR, Waltz J et al (2021) Automated detection of lung nodules and coronary artery calcium using artificial intelligence on low-dose CT scans for lung cancer screening: accuracy and prognostic value. BMC Med 19:1–14 33658025 10.1186/s12916-021-01928-3 PMC7931546

[15] Abràmoff MD, Lavin PT, Birch M, Shah N, Folk JC (2018) Pivotal trial of an autonomous AI-based diagnostic system for detection of diabetic retinopathy in primary care offices. npj Digit Med 1(1):39. 10.1038/s41746-018-0040-6 31304320 10.1038/s41746-018-0040-6 PMC6550188

[16] Compton EC, Cruz T, Andreassen M et al (2023) Developing an artificial intelligence tool to predict vocal cord pathology in primary care settings. Laryngoscope 133(8):1952–1960 36226791 10.1002/lary.30432

[17] Nakajo K, Ninomiya Y, Kondo H et al (2023) Anatomical classification of pharyngeal and laryngeal endoscopic images using artificial intelligence. Head Neck 45(6):1549–1557. 10.1002/hed.27370 37045798 10.1002/hed.27370

[18] Kim H, Jeon J, Han YJ et al (2020) Convolutional neural network classifies pathological voice change in laryngeal cancer with high accuracy. J Clin Med 9(11):3415 33113785 10.3390/jcm9113415 PMC7692693

[19] Parker F, Brodsky MB, Akst LM, Ali H (2021) Machine learning in laryngoscopy analysis: a proof of concept observational study for the identification of post-extubation ulcerations and granulomas. Ann Otol Rhinol Laryngol 130(3):286–291. 10.1177/0003489420950364 32795159 10.1177/0003489420950364

[20] Żurek M, Jasak K, Niemczyk K, Rzepakowska A (2022) Artificial intelligence in laryngeal endoscopy: systematic review and meta-analysis. J Clin Med 11(10):2752 35628878 10.3390/jcm11102752 PMC9144710

[21] Zhou X, Ma L, Brown W et al (2021) Automatic detection of head and neck squamous cell carcinoma on pathologic slides using polarized hyperspectral imaging and machine learning. In: SPIE, pp 165–173 10.1117/12.2582330 PMC8699168 34955584

[22] Ren J, Jing X, Wang J et al (2020) Automatic recognition of laryngoscopic images using a deep-learning technique. Laryngoscope 130(11):E686–E693. 10.1002/lary.28539 32068890 10.1002/lary.28539

[23] Wu Q, Wang X, Liang G et al (2023) Advances in image-based artificial intelligence in otorhinolaryngology-head and neck surgery: a systematic review. Otolaryngol-Head Neck Surg 169(5):1132–1142. 10.1002/ohn.391 37288505 10.1002/ohn.391

[24] Bensoussan Y, Vanstrum EB, Johns Iii MM, Rameau A (2023) artificial intelligence and laryngeal cancer: from screening to prognosis: a state of the art review. Otolaryngol-Head Neck Surg 168(3):319–329. 10.1177/01945998221110839 35787073 10.1177/01945998221110839

[25] Moher D, Liberati A, Tetzlaff J, Altman DG (2009) Preferred reporting items for systematic reviews and meta-analyses: the PRISMA statement. PLoS Med 6(7):e1000097. 10.1371/journal.pmed.1000097 19621072 10.1371/journal.pmed.1000097 PMC2707599

[26] OLoEW G The Oxford levels of evidence 2. Oxford Centre for Evidence- Based Medicine. Accessed March 20, 2024. https://www.cebm.ox.ac.uk/resources/levels-of-evidence/ocebm-levels-of-evidence. Accessed 20 Mar 2024

[27] Whiting PF, Rutjes AW, Westwood ME et al (2011) QUADAS-2: a revised tool for the quality assessment of diagnostic accuracy studies. Ann Intern Med 155(8):529–536. 10.7326/0003-4819-155-8-201110180-00009 22007046 10.7326/0003-4819-155-8-201110180-00009

[28] Egger M, Smith GD, Schneider M, Minder C (1997) Bias in meta-analysis detected by a simple, graphical test. BMJ 315(7109):629–634 9310563 10.1136/bmj.315.7109.629 PMC2127453

[29] Sterne JA, Egger M (2001) Funnel plots for detecting bias in meta-analysis: guidelines on choice of axis. J Clin Epidemiol 54(10):1046–1055 11576817 10.1016/s0895-4356(01)00377-8

[30] Page MJ, McKenzie JE, Bossuyt PM et al (2021) The PRISMA 2020 statement: an updated guideline for reporting systematic reviews. BMJ 372:n71. 10.1136/bmj.n71 33782057 10.1136/bmj.n71 PMC8005924

[31] Bengs M, Westermann S, Gessert N et al (2020) Spatio-spectral deep learning methods for in-vivo hyperspectral laryngeal cancer detection. In: SPIE, pp 369–374

[32] Cho WK, Lee YJ, Joo HA et al (2021) Diagnostic accuracies of laryngeal diseases using a convolutional neural network-based image classification system. Laryngoscope 131(11):2558–2566 34000069 10.1002/lary.29595

[33] Dunham ME, Kong KA, McWhorter AJ, Adkins LK (2022) Optical biopsy: automated classification of airway endoscopic findings using a convolutional neural network. Laryngoscope 132(S4):S1–S8. 10.1002/lary.28708 32343434 10.1002/lary.28708

[34] Esmaeili N, Illanes A, Boese A, Davaris N, Arens C, Friebe M (2019) Novel automated vessel pattern characterization of larynx contact endoscopic video images. Int J Comput Assist Radiol Surg 14(10):1751–1761. 10.1007/s11548-019-02034-9 31352673 10.1007/s11548-019-02034-9 PMC6797664

[35] Esmaeili N, Sharaf E, Gomes Ataide EJ et al (2021) Deep convolution neural network for laryngeal cancer classification on contact endoscopy-narrow band imaging. Sensors (Basel). 10.3390/s21238157 34884166 10.3390/s21238157 PMC8662427

[36] Inaba A, Hori K, Yoda Y et al (2020) Artificial intelligence system for detecting superficial laryngopharyngeal cancer with high efficiency of deep learning. Head Neck 42(9):2581–2592. 10.1002/hed.26313 32542892 10.1002/hed.26313

[37] Moccia S, De Momi E, Guarnaschelli M et al (2017) Confident texture-based laryngeal tissue classification for early stage diagnosis support. J Med Imaging (Bellingham) 4(3):034502. 10.1117/1.Jmi.4.3.034502 28983494 10.1117/1.JMI.4.3.034502 PMC5621380

[38] Turkmen HI, Karsligil ME, Kocak I (2015) Classification of laryngeal disorders based on shape and vascular defects of vocal folds. Comput Biol Med 62:76–85 25912989 10.1016/j.compbiomed.2015.02.001

[39] Xiong H, Lin P, Yu JG et al (2019) Computer-aided diagnosis of laryngeal cancer via deep learning based on laryngoscopic images. EBioMedicine 48:92–99. 10.1016/j.ebiom.2019.08.075 31594753 10.1016/j.ebiom.2019.08.075 PMC6838439

[40] Yan P, Li S, Zhou Z et al (2023) Automated detection of glottic laryngeal carcinoma in laryngoscopic images from a multicentre database using a convolutional neural network. Clin Otolaryngol 48(3):436–441. 10.1111/coa.14029 36624555 10.1111/coa.14029

[41] Wu F, Wu P, Hou Y, Shang H (2021) Neural network for image classification of laryngeal cancer. In: 2021 International conference on networking systems of AI (INSAI), Shanghai, China, pp 239–243

[42] Li Z, Li Z, Chen Q et al (2022) Machine-learning-assisted spontaneous Raman spectroscopy classification and feature extraction for the diagnosis of human laryngeal cancer. Comput Biol Med 146:105617. 10.1016/j.compbiomed.2022.105617 35605486 10.1016/j.compbiomed.2022.105617

[43] Zhang L, Wu Y, Zheng B et al (2019) Rapid histology of laryngeal squamous cell carcinoma with deep-learning based stimulated Raman scattering microscopy. Theranostics. 9:2541–2554. 10.7150/thno.32655 31131052 10.7150/thno.32655 PMC6526002

[44] Valjarevic S, Jovanovic MB, Miladinovic N et al (2023) Gray-level co-occurrence matrix analysis of nuclear textural patterns in laryngeal squamous cell carcinoma: focus on artificial intelligence methods. Microsc Microanal 29(3):1220–1227. 10.1093/micmic/ozad042 37749686 10.1093/micmic/ozad042

[45] Wang CT, Chuang ZY, Hung CH, Tsao Y, Fang SH (2022) Detection of glottic neoplasm based on voice signals using deep neural networks. IEEE Sensors Letters 6(3):1–4. 10.1109/LSENS.2022.3152738

[46] Siegel RL, Miller KD, Wagle NS, Jemal A (2023) Cancer statistics, 2023. CA Cancer J Clin 73(1):17–48. 10.3322/caac.21763 36633525 10.3322/caac.21763

[47] Bohr A, Memarzadeh K (2020) The rise of artificial intelligence in healthcare applications. Artif Intell Healthc 26 (**Artificial intelligence, healthcare applications, machine learning, precision medicine, ambient assisted living, natural language programming, machine vision**), pp 26–52

[48] Pinto-Coelho L (2023) How artificial intelligence is shaping medical imaging technology: a survey of innovations and applications. Bioengineering (Basel). 10.3390/bioengineering10121435 38136026 10.3390/bioengineering10121435 PMC10740686

[49] Yao P, Usman M, Chen YH et al (2022) Applications of artificial intelligence to office laryngoscopy: a scoping review. Laryngoscope 132(10):1993–2016. 10.1002/lary.29886 34582043 10.1002/lary.29886

[50] Megwalu UC, Panossian H (2016) Survival outcomes in early stage laryngeal cancer. Anticancer Res 36(6):2903–2907 27272804

[51] Kim H, Jeon J, Han YJ et al (2020) Convolutional neural network classifies pathological voice change in laryngeal cancer with high accuracy. J Clin Med. 10.3390/jcm9113415 33113785 10.3390/jcm9113415 PMC7692693

[52] Hu HT, Wang W, Chen LD et al (2021) Artificial intelligence assists identifying malignant versus benign liver lesions using contrast-enhanced ultrasound. J Gastroenterol Hepatol 36(10):2875–2883. 10.1111/jgh.15522 33880797 10.1111/jgh.15522 PMC8518504

[53] Wan YL, Wu PW, Huang PC et al (2020) The use of artificial intelligence in the differentiation of malignant and benign lung nodules on computed tomograms proven by surgical pathology. Cancers (Basel). 10.3390/cancers12082211 32784681 10.3390/cancers12082211 PMC7464412

[54] Wang B, Wan Z, Li C et al (2022) Identification of benign and malignant thyroid nodules based on dynamic AI ultrasound intelligent auxiliary diagnosis system. Front Endocrinol 13:1047–1059 10.3389/fendo.2022.1018321 PMC9551607 36237194

[55] Kim J, Kim HJ, Kim C, Kim WH (2021) Artificial intelligence in breast ultrasonography. Ultrasonography 40(2):183–190. 10.14366/usg.20117 33430577 10.14366/usg.20117 PMC7994743

[56] Sampieri C, Baldini C, Azam MA et al (2023) Artificial intelligence for upper aerodigestive tract endoscopy and laryngoscopy: a guide for physicians and state-of-the-art review. Otolaryngol-Head Neck Surg 169(4):811–829. 10.1002/ohn.343 37051892 10.1002/ohn.343

[57] Dunham ME, Kong KA, McWhorter AJ, Adkins LK (2022) Optical biopsy: automated classification of airway endoscopic findings using a convolutional neural network. Laryngoscope 132(Suppl 4):S1-s8. 10.1002/lary.28708 32343434 10.1002/lary.28708

[58] Nogués-Sabaté A, Aviles-Jurado FX, Ruiz-Sevilla L et al (2018) Intra and interobserver agreement of narrow band imaging for the detection of head and neck tumors. Eur Arch Otorhinolaryngol 275:2349–2354 30019190 10.1007/s00405-018-5063-8

