# Supplementary material for: Application of artificial intelligence in laryngeal lesions: a systematic review and meta-analysis
Source: Eur Arch Otorhinolaryngol. 2024 Nov 22;282(3):1543–55. doi: 10.1007/s00405-024-09075-0 (PMC11890366; doi:10.1007/s00405-024-09075-0)
Supplement: Supplementary file 1 — Supplementary file1 (DOCX 1011 KB) [file 405_2024_9075_MOESM1_ESM.docx]

**Table S1:**Search Terms

| Database | Search Terms |
| --- | --- |
| Pubmed | (("Artificial Intelligence"[Mesh]) OR "artificial intelligence" OR "a.i." OR "machine learning" OR radiomics OR "deep learning") AND (("Laryngeal Neoplasms"[Mesh]) OR "laryngeal cancer" OR "laryngeal carcinoma" OR Larynx OR voice OR "laryngeal neoplasm" OR "glottic cancer" OR "glottic carcinoma" OR "glottic neoplasm") |
| SCOPUS | ( TITLE-ABS-KEY ( ( "Artificial Intelligence") OR "artificial intelligence" OR "a.i." OR "machine learning" OR radiomics OR "deep learning" ) AND TITLE-ABS-KEY ( ( "Laryngeal Neoplasms" [mesh] ) OR "glottic cancer" OR "glottic carcinoma" OR "glottic neoplasm" OR "laryngeal cancer" OR "laryngeal carcinoma" OR "laryngeal neoplasm" ) ) |
| CINAHL Complete | (("Artificial Intelligence") OR "artificial intelligence" OR "a.i." OR "machine learning" OR radiomics OR "deep learning") AND (("Laryngeal Neoplasms"[Mesh]) OR "laryngeal cancer" OR "laryngeal carcinoma" OR "laryngeal neoplasm" OR "glottic cancer" OR "glottic carcinoma" OR "glottic neoplasm") - 18 |
| Cochrane | ("artificial intelligence" OR "a.i." OR "machine learning" OR radiomics OR "deep learning") AND ("laryngeal cancer" OR "laryngeal carcinoma" OR "laryngeal neoplasm" OR "glottic cancer" OR "glottic carcinoma" OR "glottic neoplasm") |


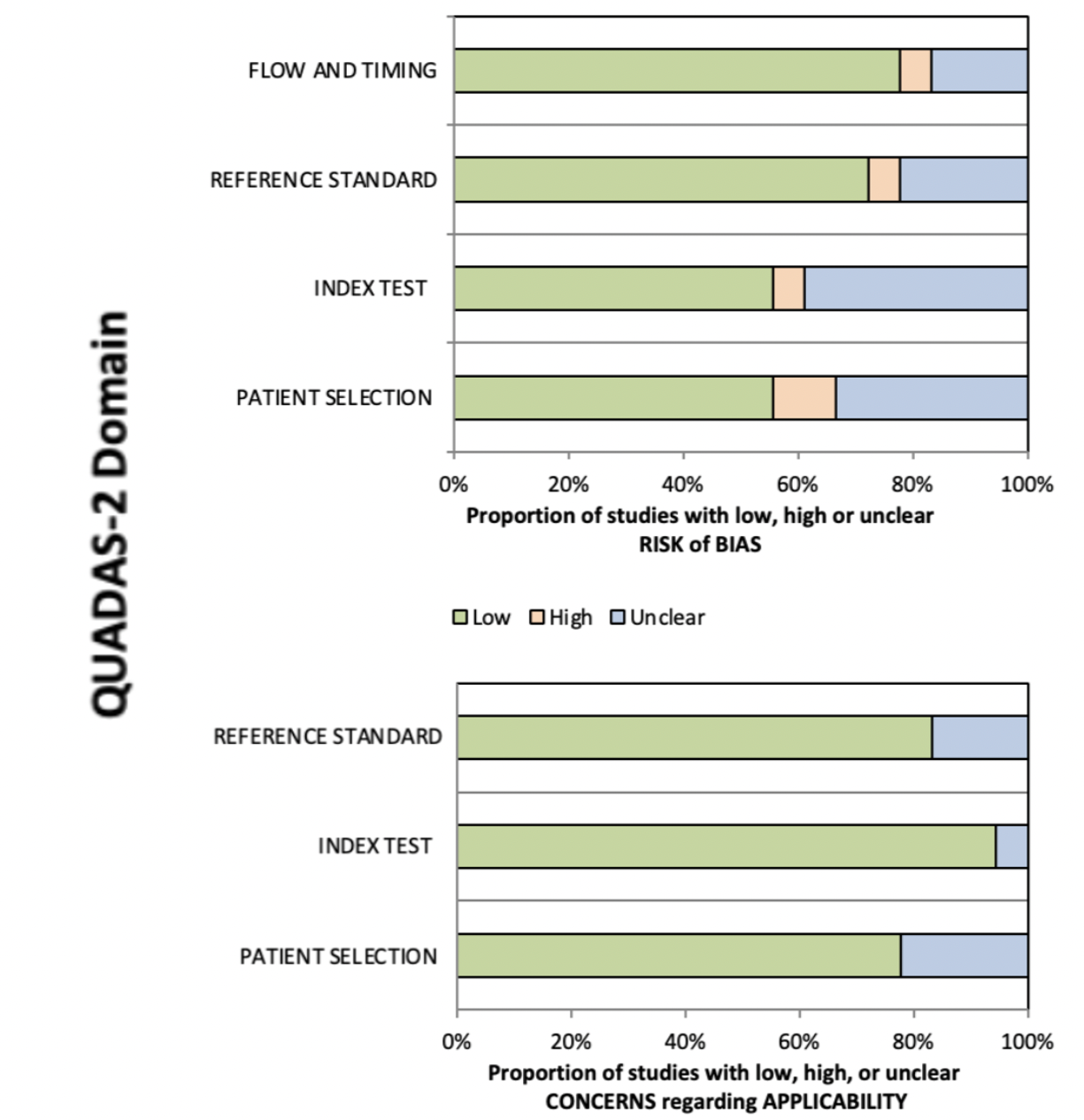


**Figure S1.** QUADAS-2 Risk of Bias.


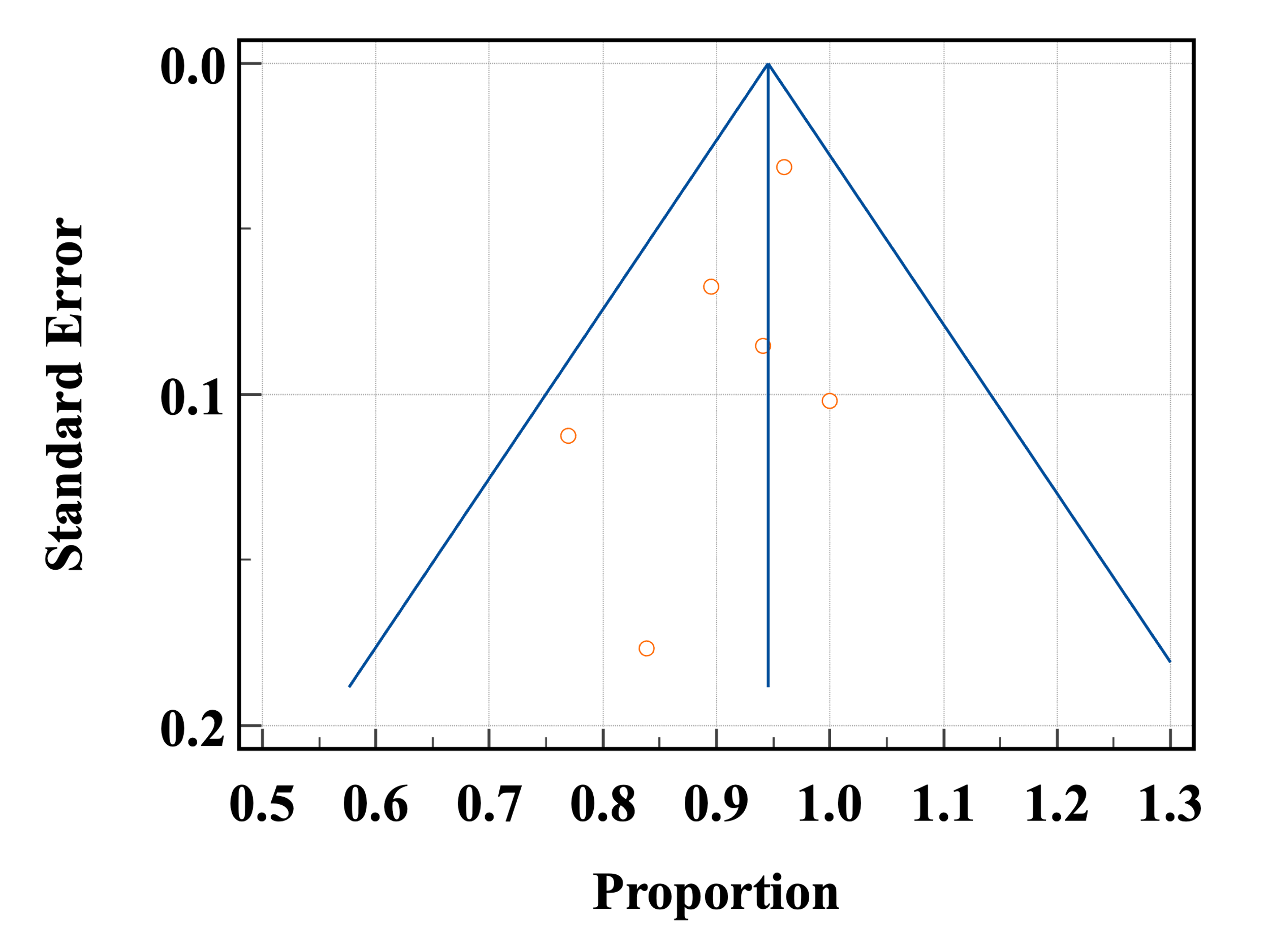


**Figure S2:** Funnel Plot
